# The Impact of Transformational Leadership on Employee Retention: Mediation and Moderation Through Organizational Citizenship Behavior and Communication

**DOI:** 10.3389/fpsyg.2020.00314

**Published:** 2020-03-17

**Authors:** Hongyun Tian, Shuja Iqbal, Shamim Akhtar, Sikandar Ali Qalati, Farooq Anwar, Muhammad Aamir Shafique Khan

**Affiliations:** ^1^School of Management, Jiangsu University, Zhenjiang, China; ^2^Lahore Business School, The University of Lahore, Lahore, Pakistan

**Keywords:** transformational leadership, organizational citizenship behavior, communication, employee retention, small- and medium-sized enterprises

## Abstract

This study investigates the impact of transformational leadership on employee retention in small- and medium-sized enterprises (SMEs) and probes the mediating role of organizational citizenship behavior (OCB) and the moderating role of communication. Data were collected using convenience sampling from 505 employees of SMEs. A Smart PLS structural equation modeling (PLS-SEM) was used to estimate the various relationships. The findings of the study reveal a positive and significant relationship between transformational leadership and OCB. Similarly, this study finds a positive and significant relationship in OCB and employee retention. In addition, OCB had a positive mediating effect on the relationship between transformational leadership and employee retention. Furthermore, communication positively moderates the transformational leadership– OCB and OCB–employee retention relationships. Leaders at SMEs should implement the traits of transformational leadership such as developing a compelling vision for employees, focusing on goal achievement, having problem-solving techniques, having a sense of purpose, and spending time on the training and development of the team to enhance OCB and employee retention.

## Introduction

Small- and medium-sized enterprises (SMEs) face high levels of uncertainty and complexity concerning employee retention (ER) (Park et al., 2019). Leadership plays a vital role in retaining employees (Covella et al., 2017) and enhancing organizational citizenship behavior (OCB) (Ahmet, 2014). Several leadership styles, including transactional, instrumental, laissez-faire, and transformational leadership (TL), have been studied in recent years (Antonakis and House, 2014). TL inspires followers by attraction to advanced moral values and ideas (Burns, 1978a). In contrast to transactional leadership, TL significantly increases employee commitment within the organization (Deichmann and Stam, 2015). Against the background of Bass (1985), the TL theory best explains the model constructed for this study. The theory supports the idea that transformational leaders modify the behavior of subordinates (Burns, 1978a), resulting in a higher ER (Sow et al., 2016). Furthermore, TL increases the intellectual ability of employees (Fletcher et al., 2019). Past research shows that several of the world’s most successful companies have achieved their goals by implementing the TL process (Sow et al., 2016; Dedaj, 2017; Jiang et al., 2017; Maaitah, 2018).

Employee retention is essential if organizations are to achieve and maintain success (Das and Baruah, 2013; Arachchillage and Senevirathna, 2017; Paul and Vincent, 2018). ER has always been a significant concern for organizations because experienced employees make vital contributions to the success of an organization (Das and Baruah, 2013). Additionally, ER fundamentally impacts the longevity of organizations, even though it is a challenging task in this age of intense competition (Das and Baruah, 2013; Arachchillage and Senevirathna, 2017; Kaur, 2017; Nelms, 2018; Sulamuthu and Yusof, 2018). Scholars have discussed two levels of retention: individual and group turnover (Muir and Li, 2014). Therefore, leaders must eliminate the reasons for low ER with the help of the human resource management department (Deshwal, 2015; Juneja, 2015). Some scholars argue that TL plays a vital role in ER (Khan, 2015; Kossivi et al., 2016; Nasir and Mahmood, 2016; Gyensare et al., 2017) and achievement of personal and organizational goals (Sow et al., 2016; Gyensare et al., 2017). Dimensions of TL, including “idealized influence, inspirational motivation, intellectual stimulation, and individualized consideration,” affect employee performance and retention (Jiang et al., 2017).

It is essential to note that leaders’ effective communication and motivation enhance employee satisfaction (Sergeeva, 2018), OCB (Yildirim, 2014; Herfina and Rubini, 2015; Chan and Lai, 2017), which significantly affects ER (Paillé et al., 2015; Popescu et al., 2015; Olendo and Muindi, 2017). There is evidence that employees show OCB when they are in an optimistic mood, and this finding has been further tested by relational mechanisms (Nohe and Hertel, 2017). The relationship between TL and OCB is based on the trust between leaders and employees (Nohe and Hertel, 2017). Owing to the direct and indirect impacts of interlinked behaviors, the effects of OCB on the ER cannot be ignored. Individual OCB affects ER in a way that the more the employees show individual OCB and a macrointerest in an organization, the less they will leave the organizations. Moreover, organizational factors such as helping, civic virtue, and sportsmanship affect the OCB of employees, further affecting ER (Paillé et al., 2015). Past studies on TL have examined the mediating role of OCB on sustainable employee performance (Jiang et al., 2017), creativity, and ER (Rashid et al., 2018). However, this study aims to examine the mediating role of OCB between TL and ER. The lower level of ER in SMEs can be managed with proper communication, which leads to higher ER and SME performance (Ugbam et al., 2012; Effiong et al., 2017). However, there is little research investigating the main reasons for high turnover in SMEs: that turnover is mainly due to their spending fewer resources on the well-being of employees compared with large organizations (Bilau et al., 2015).

This study has the following contributions. Our approach takes a more inclusive perception to indulge the complex mediation role of OCB on ER. Previous studies on TL mainly examined the mediation role of OCB on sustainable employee performance (Jiang et al., 2017), creativity, and ER (Khokhar and Zia-ur-Rehman, 2017). However, we examine the mediating role of OCB on ER in Chinese SMEs. Furthermore, previous studies on TL examined the positive effects of communication on employee output and efficacy (Hills, 2015; Luthra and Dahiya, 2015; Sadia et al., 2016). Moreover, effective communication by leaders was observed as a significant antecedent of OCB (Yildirim, 2014; Herfina and Rubini, 2015; Diebig et al., 2017), but the moderating role of communication on TL, OCB, and ER was overlooked. We examine the moderation mechanism of communication. Finally, our study enriches the literature about TL, OCB, and ER.

## Theoretical Background and Hypothesis

Past research has examined employees’ behavior predicted by several factors, such as the creation of a positive organizational climate to stimulate safe work behavior in employees (Smith-Crowe et al., 2003). Employees’ behavior, including OCB, was positively affected by the ethical climate through the social identity approach (Pagliaro et al., 2018). Ethical climates such as friendship utilizing the social identity approach projected better behaviors and attitudes of employees concerning many outcomes including turnover intention (Teresi et al., 2019). Studies also found that organizational justice theory impacts the effects on OCB through perceived restorativeness (Bellini et al., 2019). However, studies on TL have examined TL theory and its four dimensions significantly affecting OCB (Jiang et al., 2017) and ER (Adekanbi, 2016; Sow et al., 2016). It is based on the view that transformational leaders transform their followers by changing their insights, ambitions, morals, and potential (Bass, 1985). The qualities of leaders stimulate change, and they interconnect and establish ways of change to achieve the desired results (Burns, 1978b). The original theory of Burns says that leaders can change the life of the subordinates by changing their ambitions, insights, values, and expectations. Based on the Bass (1985) theory, the independent variable TL in this study is linked to four factors, including *individual consideration* (IC), which refers to the concept that the needs of the members of the team are focused and prioritized. The leader serves as an exemplar, counselor, organizer, and trainer to encourage an employee to take part in team activities and exhibit OCB. *Intellectual stimulation* (IS) includes support and encouragement provided by managers or leaders to members of the team, to generate innovative ideas on how to change existing procedures or orders in order to produce effective results; this, in turn, helps to boost ER. *Leader inspiration* (LI) involves helping followers to pursue a goal. Leaders establish and convey a vision or objective that they want the team to achieve, and the team is inspired to achieve that goal thanks to the leader’s explanation of the reasons for doing so. The leaders help and coach their team members to proceed in achieving their tasks. *Idealized influence* (II) includes setting a practical example as a leader and exhibiting the qualities of innovative thinking, trust, uprightness, faith, interest, pride, and effective communication (Bass, 1985). These factors significantly affect ER in SMEs.

### Transformational Leadership

Transformational leadership focuses on real-time problems, defines new benchmarks, builds understanding, and motivates and shapes the behavior of subordinates to achieve organizational goals effectively (Manshadi et al., 2014; Nagy and Edelman, 2014; Middleton et al., 2015; Jiang et al., 2017; Matwally and El Zarka, 2017; Arif and Akram, 2018). Studies suggest that the role of every manager in the organization is to be a leader instead of only a manager (Hall et al., 2015). Organizational success improves through the enhanced effects of TL (Sun and Henderson, 2017; Maaitah, 2018). TL also enhances employee performance in groups/teams (Amin et al., 2016). Past research has shown that TL plays a critical role in the success of Chinese SMEs (Lin and Sun, 2018). Chinese employees prefer leaders who exhibit the traits of TL (idealized consideration, IS, LI, and II), for instance, acting as a role model, selflessness (Farh and Cheng, 2000), avoiding the use of abusive power, setting a good example, and working for employee well-being (Dunfee and Warren, 2001; Cheng et al., 2004; Xiaoxia and Jing, 2006; Chen et al., 2012; Lin and Sun, 2018; Su et al., 2019).

During the past decade, there has been extensive research on TL and its relationship to multiple outcomes. TL significantly affects OCB (Rodrigues and Ferreira, 2015; Sarwar et al., 2015; Ismaeelzadeh et al., 2016; Saif et al., 2016; Cofie, 2018; Hassi, 2018). Additionally, employees exceed their assigned duties when a TL style is used (OCB, 2018). Within SMEs, all four dimensions of TL play a vital role in cultivating OCB (Jiang et al., 2017). Past research examined the positive effect of TL traits on OCB (Emami et al., 2012; Pickford and Joy, 2016; Majeed et al., 2017; Cofie, 2018). Bass (1985) explained that the qualities of transformational leaders such as individualized consideration, IS, inspirational motivation, and individualized influence enhance OCB in employees; for instance, Jiang et al. (2017) examined positive impacts of TL dimension on OCB. The following relationship has been established for this study:

Hypothesis 1: TL has a positive and significant impact on OCB.

### Employee Retention

There is a considerable amount of literature on ER, highlighting its importance for all types of firms. The cost of losing employees is much higher than retaining them through compensation plans (Carter et al., 2019). To investigate this issue, many factors have been considered, such as the control variables of age, education, experience, sex (Deshwal, 2015), peer support (Ali et al., 2017), recruitment and selection, job preview, organizational culture, employee relations, awards and recognition, work–life balance, and training and development (Olendo and Muindi, 2017). Leadership equally benefits employees and organizations, and specifically, TL affects ER (Amankwaa and Anku-Tsede, 2015), both directly and indirectly (Khan, 2015; Nohe and Hertel, 2017). Transformational leaders improve subordinates’ performance by achieving organizational goals (Sow et al., 2016) and implementing a reward system to retain employees (Adekanbi, 2016). TL increases ER (Abouraia and Othman, 2017; Gyensare et al., 2017; Jiang et al., 2017) and reduces turnover intention (Maaitah, 2018).

Transformational leadership influences the retention choices of employees (Sulamuthu and Yusof, 2018). Furthermore, the theory of transformational leaders strongly supports the relationship between TL and ER (Amankwaa and Anku-Tsede, 2015; Khan, 2015). This study proposes that employees show higher levels of retention when leaders exhibit individualized influence, IS, inspirational motivation, and individualized influence. Past studies have examined the relationship between TL and ER through TL theory (Adekanbi, 2016; Sow et al., 2016). Therefore, we have proposed the second hypothesis, as follows:

Hypothesis 2: TL has a positive and significant impact on ER.

### Organizational Citizenship Behavior

The concept of OCB first appeared in the early 1980s and initially described the specified behavior of employees within the organizations. Scholars described “organizational commitment and individual traits” as factors to enhance OCB (Emami et al., 2012). Voluntary behaviors of employees to prove themselves as good citizens of the organization are called OCB (Tambe, 2014). Similarly, an organization’s success is critical without OCB (Obiora and Okpu, 2014). The positive effects of OCB’s three dimensions, namely, public benefits, sportsmanship, and self-sacrifice, on employee well-being increase ER (Tambe and Shanker, 2015; Yurcu et al., 2015). Additionally, OCB refers to the behaviors that employees exhibit outside of their formal responsibilities. Leaders can help employees enhance OCB and benefit the organization (Pickford and Joy, 2016; Yaylaci, 2016; Zeyada, 2018). Furthermore, OCB refers to discretionary behavior, which is not directly or explicitly recognized by the formal reward system. However, such behaviors promote the effective functioning of the organization (Majeed et al., 2017). OCB enhances both individual and team performance (Mehdizadeh et al., 2018; OCB, 2018). Few studies have also examined a negative relationship between OCB and turnover intention (Islam et al., 2012). A higher level of OCB significantly affects ER (Dash and Pradhan, 2014; Paillé et al., 2015; Pivi and Hassan, 2015; Anvari et al., 2017; Olendo and Muindi, 2017; Mittal and Kaur, 2018). This relationship will be analyzed in Chinese SMEs with the following hypothesis:

Hypothesis 3: OCB has a positive and significant impact on ER.

### Mediation Effect of Organizational Citizenship Behavior

Scholars have argued that OCB plays a critical role in SMEs’ success in China (Farh et al., 2004). TL affects ER in Chinese SMEs, both directly and indirectly (Sun and Wang, 2017). Furthermore, OCB was found to partially mediate the relationship between internal corporate social responsibility and intention to quit (Rashid et al., 2018). Jiang et al. (2017) found that OCB mediated more than half of the effects of TL on sustainable employee performance. Saoula and Johari (2016) studied the positive mediation of OCB between the relationship of perceived organizational support and turnover intention.

Similarly, Khokhar and Zia-ur-Rehman (2017) examined the positive and significant mediating role of OCB between TL, creativity, and ER. Selamat and Ran (2019) found that OCB significantly mediates the relationship between organizational justice and performance within SMEs in China. Chiang and Hsieh (2012) found that OCB partially mediated the relationship between perceived organizational support and job performance. The four traits of Bass’s (1985) theory, including individualized influence, IS, inspirational motivation, and individualized consideration, develop specific leadership skills in leaders. Transformational leaders help to develop OCB in their followers (Emami et al., 2012; Majeed et al., 2017; Cofie, 2018), further leading to ER (Adekanbi, 2016; Sow et al., 2016). However, this relationship with the perspective of Chinese SMEs has not been extensively explored. We propose that OCB plays a mediating role in the relationship between TL and ER. Therefore, we develop the following relationship to check the mediation of OCB:

Hypothesis 4: OCB positively mediates the relationship between TL and ER.

### Moderation Effect of Communication

Communication is critical to motivate employees, measure success, deliver products and services to customers (Conrad, 2014; Nwata et al., 2016), and enhance the performance of employees (Femi, 2014). Effective communication between leaders and employees significantly increases employee output and efficiency (Hills, 2015; Luthra and Dahiya, 2015; Sadia et al., 2016) and employee commitment (Marchalina and Ahmad, 2017). Therefore, through effective communication, employees feel valued, motivated, and rewarded for their efforts toward organizational success (Kukla, 2017), and individual and organizational betterment (Sergeeva, 2018). According to scholars, “communicators have finally started putting a greater focus on the development of leadership and management communication” (Gatehouse, 2018).

**FIGURE 1:**
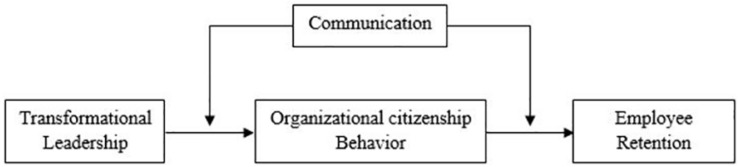
Research framework.

Scholars have argued that effective communication by leaders increases OCB (Yildirim, 2014; Herfina and Rubini, 2015). Diebig et al. (2017) studied the positive moderation effect of direct communication on TL and daily team cooperation. Garnett et al. (2008) studied communication as a moderator between organizational culture and public organizations’ performance. Pettit et al. (1997) studied the moderation of communication between job performance and satisfaction. Similarly, Villegas and Cerveny (2004) also found communication to mediate between job satisfaction and absenteeism positively. On the basis of the discussion above, we propose that communication could be considered a moderator between the relationships of TL, OCB, and ER. Bass’s (1985) theory supports the idea that leaders must effectively convey the vision and mission they have for the organization. This study explores the relationship in SMEs in China. All the constructed relationships are presented in Figure 1.

Hypothesis 5: Communication positively moderates the relationship between TL and OCB.

Hypothesis 6: Communication positively moderates the relationship between OCB and ER.

## Materials and Methods

### Sample and Procedure

Employees of manufacturing SMEs in Jiangsu Province Mainland China participated in this study. A sample size of 505 was obtained using random sampling technique. A total of 600 employees were contacted personally and online to distribute the questionnaires. Out of the 516 questionnaires received, 11 were rejected owing to missing information/incomplete responses. A total of 505 (84.16%) response rates were recorded for further examination. A total of 406 male and 98 female participants accounted for 80 and 20%, respectively. Participants in the 20–30 (210), 31–40 (231), 41–50 (42), and 51–60 (21) age groups accounted for 41.5, 46, 8, and 4%, respectively. Similarly, 56 participants had intermediate (high school) education, 210 participants had bachelor’s degrees, 210 participants had master’s degrees, and 28 participants had Ph.D. degrees, accounting for 11, 41.5, 41.5, and 5.5%, respectively. Twenty-eight participants had less than 1 year of work experience, 196 participants had 1–5 years, 182 participants had 6–10 years, 77 participants had 11–15 years, and 21 participants had more than 15 years of work experience, accounting for 5.5, 39, 36, 15, and 4% of the total participants, respectively (Table 1).

**TABLE 1:** Demographic information.

Controls		Variance
Gender	Male	406(80%)
	Female	98(20%)
Age	20–30 years	210(41.5%)
	31–40 years	231(46%)
	41–50 years	42(8%)
	51–60 years	21(4%)
Career level	Entry level	119(23.5%)
	Middle level	294(58%)
	High level	91(18%)
Education	High school	56(11%)
	Bachelors	210(41.5%)
	Masters	210(41.5%)
	Ph.D.	28(5.5%)
Experience	<1 year	28(5.5%)
	1–5 years	196(39%)
	6–10 years	182(36%)
	11–15 years	77(15%)
	>15 years	21(4%)

### Measures

#### Transformational Leadership

This study examined the TL (e.g., “My leader articulates a compelling vision”) by five-item scale (α = 0.931, Table 2), developed by Bass and Avolio (1995).

**TABLE 2:** Measurement model.

Construct	Item code	Loading	*p*-value	CA	CR	AVE
Transformational leadership				0.931	0.948	0.785

	TL1	0.886	<0.000			
	TL2	0.888	<0.000			
	TL3	0.911	<0.000			
	TL4	0.881	<0.000			
	TL5	0.863	<0.000			

Employee retention				0.926	0.944	0.772

	ER1	0.846	<0.000			
	ER2	0.875	<0.000			
	ER3	0.894	<0.000			
	ER4	0.904	<0.000			
	ER5	0.873	<0.000			

Organizational citizenship behavior				0.897	0.924	0.709

	OCB1	0.859	<0.000			
	OCB2	0.849	<0.000			
	OCB3	0.907	<0.000			
	OCB4	0.850	<0.000			
	OCB5	0.737	<0.000			

Communication				0.895	0.923	0.705

	C1	0.846	<0.000			
	C2	0.880	<0.000			
	C3	0.815	<0.000			
	C4	0.846	<0.000			
	C5	0.810	<0.000			

*CA, Cronbach’s alpha; CR, composite reliability; AVE, average variance extracted.*

#### Organizational Citizenship Behavior

Five items adapted from Lee and Allen (2002) were used to measure OCB (e.g., “I show genuine concern and courtesy toward colleagues,” α = 0.897, Table 2).

#### Employee Retention

This study measured ER (e.g., “My work gives me satisfaction in this company”) by five-item scale (α = 0.926, Table 2), developed by Kyndt et al. (2009).

#### Communication

Five items adapted from Roberts and O’Reilly (1974) were used to measure communication (e.g., “It is very important for me to progress upward in my present organization,” α = 0.895, Table 2).

### Data Analysis

The statistical software SmartPLS was used to analyze data. First, measurement model techniques were used to test the Cronbach alpha, heterotrait–monotrait (HTMT) ratio, composite reliability (CR), and average variance extracted (AVE). Second, this study used discriminant validity and correlation to analyze the theoretical model. Third, the study assessed the structural model by analyzing collinearity/common method bias [variance inflation factor (VIF)], coefficient of determination (*R*^2^), *F*^2^, predictive relevance (*Q*^2^), and standardized root mean square residual (SRMR). Finally, this study performed structural equation modeling (SEM) to test the hypothesis.

## Results

### Measurement Model

The reliability of the scales was determined by Cronbach’s alpha (CA) test. The validity of the measurement scales was found to be significant, with values of 0.895 for C, 0.926 for ER, 0.897 for OCB, and 0.931 for TL. Adequate CR or internal consistency reliability measured in the present study ranged between 0.923 and 0.948 (equal or above 0.7, as suggested by Bagozzi and Yi, 1988; Hair et al., 2011). Moreover, the present study met the threshold of convergent validity (AVE) of at least 0.50 (Fornell and Larcker, 1981; Chin, 1998; Table 2).

According to scholars, the HTMT, to assess multicollinearity within the data, should not be higher than 0.9 (Gold et al., 2001; Teo et al., 2008). The study met the standard, as results were found in the range of 0.343 to 0.736 (Table 3). The discriminant validity results are presented in Table 4, which shows a significant value of 0.84 for C, 0.879 for ER, 0.842 for OCB, and 0.886 for TL. Moreover, the results show that TL has a positive correlation with OCB (0.533), TL and ER (0.557), and OCB and ER (0.457); communication and TL, OCB, and ER have positive correlations with values of 0.59, 0.659, and 0.547, respectively.

**TABLE 3:** HTMT (heterotrait–monotrait ratio).

	C	ER	OCB	OCB*C	TL
ER	0.597				
OCB	0.736	0.49			
OCB*C	0.616	0.253	0.685		
TL	0.643	0.596	0.591	0.471	
TL*C	0.527	0.353	0.567	0.658	0.572

**TABLE 4:** Discriminant validity (latent variable correlation and square root of AVE).

	C	ER	OCB	TL
C	*0.84*			
ER	0.547	*0.879*		
OCB	0.659	0.457	*0.842*	
TL	0.590	0.557	0.553	*0.886*

*AVE, average variance extracted. The values in italics show the square root of AVE, which is greater than the correlation in the latent variable.*

### Assessment of Structural Model

This study measured collinearity and common method bias issues through the VIF. VIF is defined as the reciprocal of tolerance. As suggested by the scholars Kock (2015) and Hair et al. (2011), this study was considered bias-free with no values equal to or lower than 3.3 (Table 5). Furthermore, Harman’s single factor test (suggested by Podsakoff et al., 2003) indicated that the maximum variance that is explained by a single factor is 38.4%. Henceforth, we conclude that this dataset does not suffer from common method bias (Kock, 2015).

**TABLE 5:** Structured model results.

**Construct**	***R*^2^**	**Adj. *R*^2^**	***F*^2^**	***Q*^2^**	**VIF**	**SRMR**
ER	0.418	0.413		0.274		0.065
OCB	0.504	0.501	0.024	0.309	2.268	
C			0.104		2.152	
OCB*C			0.055		1.855	
TL			0.131		1.661	

*VIF, variance inflation factor; SRMR, standardized root mean square residual.*

According to previous studies, *R*^2^ measures the model’s predictive power (Sarstedt et al., 2014). The value of 0.418 indicates that 41.8% of variations in ER occurred because of independent variables (0.75 = substantial, 0.5 = moderate, and 0.25 = weak, as suggested by Henseler et al., 2009; Hair et al., 2011). Additionally, Cohen (2013) noted that the values of 0.02, 0.15, and 0.35 represent small, medium, and significant effects, respectively. If the value of *f*^2^ is <0.02, it indicates that there is no effect. The results of the study shown in Table 5 show that there was an effect.

Predictive relevance is an indicator of the model’s out-of-sample predictive power or predictive relevance given by Stone and Geisser’s *Q*^2^ value (Geisser, 1974; Stone, 1974). In the SEM, *Q*^2^ values larger than zero for a specific reflective endogenous latent variable indicate the path model’s predictive relevance for a particular dependent construct. The results of this study show medium predictive importance (0.02 = small, 0.15 = medium, and 0.35 = immense, as suggested by Geisser, 1974; Stone, 1974).

Standardized root mean square residual is the absolute measure of fit, and a value of zero indicates the perfect fit. SRMR is defined as “the root mean square discrepancy between the observed correlations and the model-implied correlations.” The results show a significant value of 0.065 (Table 5), and if the value of SRMR is less than 0.08, it is generally considered a good fit (Hu and Bentler, 1998). This study satisfies and ensures the goodness of fit.

### Structural Equation Modeling

**FIGURE 2:**
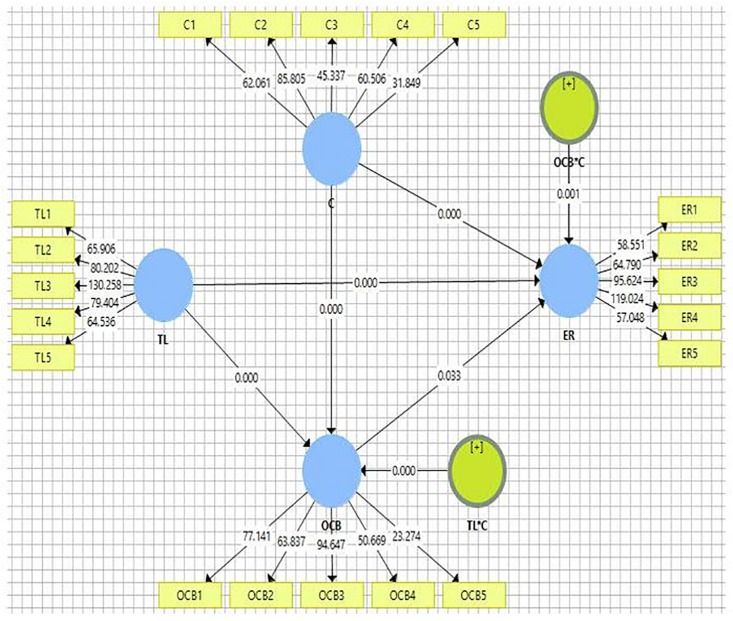
Partial Lease Square SEM model.

This study conducts the PLS-SEM to test the theoretical model. The findings show (H1) that TL had a positive and significant direct impact on OCB (β = 0.169, *t* = 4.737, *p* < 0.000). The direct effects of TL on ER (H2) were also positive and significant (β = 0.356, *t* = 6.479, *p* < 0.000). Similarly, the direct impact of OCB or ER (H3) was positive and significant (β = 0.179, *t* = 2.203, *p* = 0.033). OCB as a mediator (H4) had a positive and significant direct impact on the relationship between TL and ER (β = 0.030, *t* = 2.169, *p* = 0.000). Similarly, the impacts of communication as moderator on the relationship of TL and ER (H5), OCB, and ER were found to be positive and significant (β = 0.183, *t* = 5.035, *p* = 0.000), and H6 was noted to be negative but statistically significant (β = −0.181, *t* = 3.373, *p* = 0.001) (Table 6 and Figure 2). The results were also supported by previous studies (Jiang et al., 2017; Khokhar and Zia-ur-Rehman, 2017; Majeed et al., 2017; Olendo and Muindi, 2017; Cofie, 2018; Maaitah, 2018; Mittal and Kaur, 2018; Sulamuthu and Yusof, 2018).

**TABLE 6:** Hypothesis constructs.

Effects	Relations	β	Mean	SD	*t*-value	*p*-value	Decision
**Direct**							
H1	TL → OCB	0.169**	0.168	0.036	4.737	0.000*	Supported
H2	TL → ER	0.356**	0.359	0.055	6.479	0.000*	Supported
H3	OCB → ER	0.179**	0.177	0.081	2.203	0.033*	Supported
**Indirect or mediating**							
H4	TL → OCB → ER	0.030**	0.029	0.014	2.169	0.000*	Supported
**Indirect or moderating**							
H5	TL*C → OCB	0.183**	0.184	0.036	5.035	0.000*	Supported
H6	OCB*C → ER	−0.181**	–0.183	0.054	3.373	0.001*	Supported

*p-value < 0.05, **p-value < 0.1.

**FIGURE 3:**
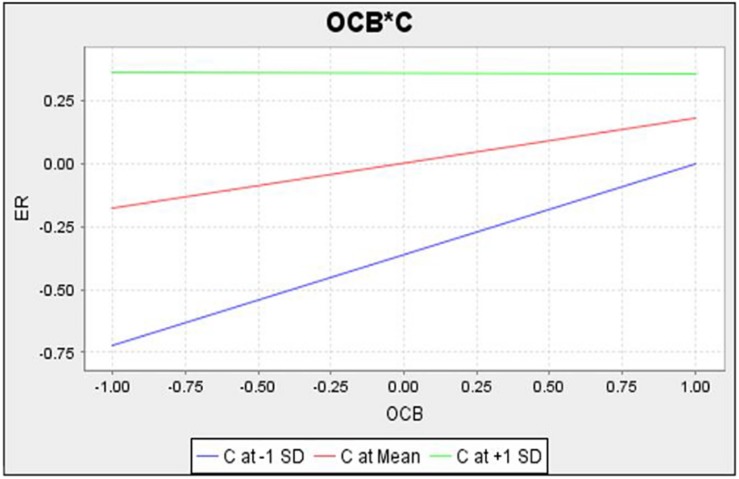
Interaction diagrame of C between ER and OCB.

**FIGURE 4:**
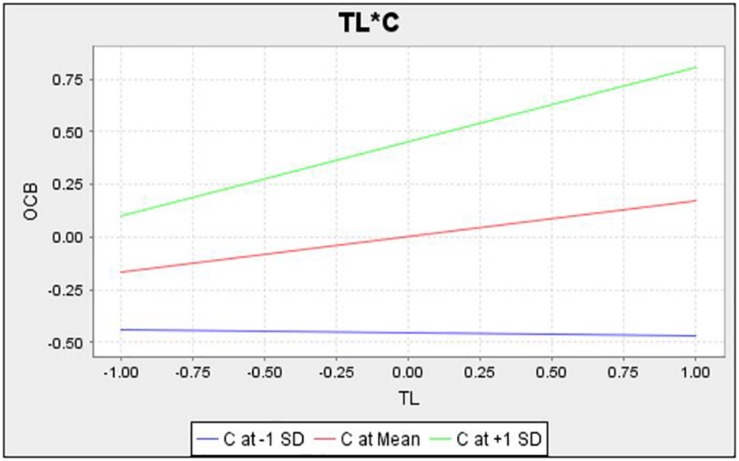
Interaction diagrame of C between OCB and TL.

Figure 3 shows the interaction of communication on the relationship between ER and OCB. The lines on the graph show that if there is highly effective communication in SMEs, the moderation effect of communication will be higher, and the ER will be increased by OCB. Similarly, the Figure 4 shows the moderation effect in the relation of OCB and TL. ER will be increased in SMEs with effective communication.

## Discussion

The present study proposed and examined a mediation model of how TL impacts ER and a moderation model of how communication impacts the relationships of TL, OCB, and ER. Consistent with this study’s predictions, the TL has a positive and significant effect on ER through the mediation of OCB. Specifically, TL enhances ER and OCB through compelling vision, goal achievement, problem solving, and training and development. This study also examined the complex moderation process of TL’s influence on OCB and OCB’s influence on ER.

### Theoretical Implications

First, the findings extend the research of TL. This study confirms that the ability of the leaders to articulate a compelling vision, skills at expressing confidence in goal achievement, innovative perspectives on problem solving, spending time on the training and development of the team, and specifically having a strong sense of purpose (Bass and Avolio, 1995) impact OCB (Rodrigues and Ferreira, 2015; Ismaeelzadeh et al., 2016; Saif et al., 2016; Majeed et al., 2017; Cofie, 2018) and ER (Amankwaa and Anku-Tsede, 2015; Khan, 2015; Jiang et al., 2017; Maaitah, 2018). This study illustrates the complexity of the relationship between OCB and ER. Results show that employees of SMEs express genuine courtesy toward coworkers, even under the most tiring business or personal situations, which helps to enhance OCB. Moreover, this study elaborates that if employees willingly help coworkers, defend the organization’s name, and express loyalty toward the organization, ER increases (Pickford and Joy, 2016; Olendo and Muindi, 2017; Mittal and Kaur, 2018).

Second, the results suggest that OCB has a decisive mediation role between TL and ER. The results show that transformational leaders can help increase ER more effectively if employees exhibit OCB (Jiang et al., 2017; Khokhar and Zia-ur-Rehman, 2017). This study has examined that communication, including the importance (to employees) of moving upward in the organization, the streams of information they communicate to their immediate boss, and their feelings about how their boss can help their career growth. This study has uniquely examined the effects of communication as a moderator between the relationships of TL, OCB, and ER. Unlike other studies, this study reveals the positive impacts of OCB as a mediator in TL and ER. The primary implication of this study is the crucial mediating role of OCB and the moderating component of communication; these findings contribute significantly to the existing literature.

### Practical and Managerial Implications

This study offers a few practical implications on how TL facilitates the increase in ER. Specifically, it is essential to understand that the traits of TL develop skills in managers to retain employees. SMEs should train leaders to develop TL characteristics in them. Accordingly, leaders should try to implement the skills of TL such as developing a compelling vision for employees, focusing on goal achievement, having problem-solving techniques, having a sense of purpose, and spending time on the training and development of the team to enhance ER.

Furthermore, leaders should pay attention to develop OCB in subordinates. Importantly, when leaders want to increase OCB, they should practice traits of TL to help and support the employee in achieving their goals, practice different problem-solving methods and train employees to enhance OCB. Moreover, leaders should not neglect the importance of communication with their employees to communicate the responsibilities clearly, to listen to the problems and issues in task performance, and to manage them accordingly.

### Limitations and Future Research Suggestions

This study has a few limitations associated. First is the time limit bounded to obtain the maximum number of responses. This study is based on cross-sectional data, and more longitudinal studies are required to develop in-depth knowledge and to capture the relationships between variables as well as to check for differences in results if longitudinal data are used instead of cross-sectional data. The data for this study were gathered from 505 employees of SMEs in China, the sample size can be increased, and comparative analysis of the same model in private and public sector organizations can be checked. Moreover, the present study is exclusively focused on SMEs in China. Applying these results to different cultural contexts and populations may require appropriate alterations. Future research may include the study of other mediating variables, such as job satisfaction, deviant workplace behavior, and supervisor conflicts. Furthermore, there are multiple approaches to analyzing the relationships between the direct and indirect paths of the model.

## Data Availability Statement

The datasets generated for this study are available on request to the corresponding author.

## Ethics Statement

This study was carried out in accordance with the recommendations of the Ethical Principles of Psychologists and Code of Conduct by the American Psychological Association’s (APA). All participants gave written informed consent in accordance with the Declaration of Helsinki. The protocol was approved by the employee’s council of the participating organizations as well as the ethics committee of Jiangsu University, Zhenjiang. The patients/participants provided their written informed consent to participate in this study.

## Author Contributions

HT, SI, and MK: conception and design of the study. SA, SI, and FA: acquisition of data and data analysis. MK and HT: performed the analysis. FA, SQ, and SI: drafting the manuscript. SA and MK: critical revision of manuscript.

## Conflict of Interest

The authors declare that the research was conducted in the absence of any commercial or financial relationships that could be construed as a potential conflict of interest.
